# Standardization of Strategies to Perform a Parafascicular Tubular Approach for the Resection of Brain Tumors in Eloquent Areas

**DOI:** 10.3390/brainsci13030498

**Published:** 2023-03-15

**Authors:** Nadin J. Abdala-Vargas, Giuseppe E. Umana, Javier G. Patiño-Gomez, Edgar Ordoñez-Rubiano, Hernando A. Cifuentes-Lobelo, Paolo Palmisciano, Gianluca Ferini, Anna Viola, Valentina Zagardo, Daniel Casanova-Martínez, Ottavio S. Tomasi, Alvaro Campero, Matias Baldoncini

**Affiliations:** 1 Neurosurgery Department, Fundación Universitaria de Ciencias de la Salud (FUCS), Hospital Infantil Universitario de San José, Cra. 19 #8A-32, Bogotá 111221, Colombia; 2 Department of Neurosurgery, Trauma and Gamma-Knife Center Cannizzaro Hospital, 95126 Catania, Italy; 3 Department of Neurosurgery, University of Cincinnati College of Medicine, Cincinnati, OH 45220, USA; 4 Department of Radiation Oncology, REM Radioterapia s.r.l., 95029 Vaigrande, Italy; 5 San Felipe Campus, Medical Faculty, University of Valparaíso, Valparaíso 2170000, Chile; 6 Department of Neurosurgery, Christian-Doppler-Klinik, Paracelsus Private Medical University, 5020 Salzburg, Austria; 7 Department of Neurological Surgery, Padilla Hospital, Tucumán T4000, Argentina; 8 Department of Neurological Surgery, San Fernando Hospital, Buenos Aires B1646, Argentina

**Keywords:** brain tumor, parafascicular tubular retractor, fiber tracking, minicraniotomy, brain mapping

## Abstract

Objective: The aim of this work is to define a methodological strategy for the minimally invasive tubular retractor (MITR) parafascicular transulcal approach (PTA) for the management of brain tumors sited in eloquent areas. Methods: An observational prospective study was designed to evaluate the benefits of PTA associated with MITRs, tractography and intraoperative cortical stimulation. They study was conducted from June 2018 to June 2021. Information regarding white matter tracts was processed, preventing a potential damage during the approach and/or resection. All patients older than 18 years who had a single brain tumor lesion were included in the study. Patients with a preoperative Karnofsky Performance Scale (KPS) score greater than 70% and a Glasgow Coma Scale (GCS) score > 14 points were included. Results: 72 patients were included in the study, the mean age was 49.6, the most affected gender was male, 12.5% presented aphasia, 11.1% presented paraphasia, 41.6% had motor deficit, 9.7% had an affection in the optic pathway, the most frequently affected region was the frontal lobe (26.3%), the most frequent lesions were high-grade gliomas (34.7%) and the measurement of the incisions was on average 5.58 cm. Of the patients, 94.4% underwent a total macroscopic resection and 90.2% did not present new postoperative neurological deficits. In all cases, a PTA was used. Conclusion: Tubular minimally invasive approaches (MIAs) allow one to perform maximal safe resection of brain tumors in eloquent areas, through small surgical corridors. Future comparative studies between traditional and minimally invasive techniques are required to further investigate the potential of these surgical nuances.

## 1. Introduction

From the first non-accidental craniotomies, in the late Paleolithic and early Neonolithic periods, to the present day, craniotomy has undergone substantial modifications. Initially, large craniotomies with great brain exposure were necessary for adequate anatomical orientation, better intra-surgical luminosity and larger surgical route considering the size of the surgical instruments and the number of people required for an intervention [1].

Similarly to the advent of microsurgery, the role of minimally invasive techniques in the field of brain neurosurgery has grown in recent decades [2]. The introduction of the neuroendoscope in the management of intracranial pathologies has been known since 1910 [3], but it was popularized only starting from 1988, when Kelly et al. described the surgical technique for the resection of brain lesions with tubular retractor and stereotaxis [4]. Some years before, Jane JA proposed the modification of the orbitozygomatic approach in the supraorbital approach, by means of a minimal craniotomy area that allowed less brain manipulation and theoretically a comparable surgical corridor, a technique which was further popularized by Reisch and Perneczky [5,6].

New surgical technologies, which include high-power lights, improved anesthetic control, high-definition microscopes, advanced imaging and stereotaxic navigation, have led to the planning of smaller, tumor-centered craniotomies that can represent lower complication rates and shorter hospital stays [7,8]. The mini-craniotomy approaches or the keyhole approaches are usually defined as a minimally invasive alternatives to a conventional craniotomy, and they offer the same advantageous approach with less brain manipulation [9]. Since its design, this approach has been partially accepted and has been implemented in the management of selected neurosurgical lesions.

Brain retraction is necessary to access skull base or intra-axial lesions and this can cause brain injury and related neurological or vascular damage [10]. At the end of the 19th century, it was clear that the excessive use of retraction, the retraction time and the uneven distribution of pressure over brain tissue play a key role for a safer brain manipulation/retraction. Thus, retraction systems have been implemented and developed like tubular retractors, and new surgical nuances have been introduced and continue to evolve nowadays. Minimally invasive tubular retractors (MITRs) are able to create a safe approach, theoretically minimizing brain damage, approach time and improving the accuracy with respect to accessing brain lesions. The aim of this study is to propose a methodological strategy for the planning of a parafascicular transulcal approach (PTA) with the help of MITRs in the management of brain tumors in eloquent areas.

## 2. Materials and Methods

### 2.1. Study Design

An observational prospective study was conducted from June 2018 to June 2021. The study was carried out after the approval of the Ethics and Research Committee of the Hospital Infantil Universitario de San José, Bogotá, Colombia. Written consent was obtained from each patient for the use and manipulation of the diagnostic and intraoperative images. The confidentiality of personal data was protected by assigning a number to each patient. All patients older than 18 years who had a single brain tumor lesion in eloquent areas were included in the study. Patients with a preoperative Karnofsky Performance Scale (KPS) score greater than 70% and a Glasgow Coma Scale (GSC) score > 14 points were included. Muscle strength was assessed in each patient using the Medical Research Council (MRC) muscle strength scale, before and after the procedure. In patients with lesions in the occipital lobe, visual acuity was evaluated by performing computerized visual campimetry. Patients were evaluated to discern whether any type of aphasia was present or not. The location of the tumors was recorded in the cerebral regions: frontal, parietal, occipital, temporal, insular, frontoparietal, parietooccipital, frontotemporal, parietotemporal and temporo-occipital. Brain tumors were classified into low-grade gliomas (LGGs), high-grade gliomas (HGGs), metastases and others.

### 2.2. Diagnostic Imaging

Gadolinium MRI data were acquired with a General Electric Signa Excite HDXT scanner (1.5T GE Healthcare, Milwaukee, WI, USA). For each patient, a presurgical brain magnetic resonance imaging (MRI) image was acquired with diffusion tensor imaging (DTI) of 140 T1 slices (1 mm thick) without GAP. After obtaining the images, they were merged and processed with the help of the neuronavigation system (Kick, Curve TM and SmartBrush, BrainLab, Munich, Germany). Information regarding white matter tracts was processed, preventing potential damage during the approach and/or resection. According to the location of the tumor, the following tracts were considered for reconstruction: Aslant’s tract, superior longitudinal fasciculus (SLF), arcuate fasciculus, inferior longitudinal fasciculus (ILF), uncinate fasciculus (UF), geniculo-calcarine fasciculus (GCT), corticospinal tract (CST) and thalamocortical tract. A Gadolinium MRI scan and a CT with contrast were performed 24–48 h postoperatively in each case.

### 2.3. Incision, Craniotomy and Extent of Resection

The size of the incision was determined intraoperatively with the help of a ruler. The size of the craniotomy was measured on its diameter using the bone algorithm of the postoperative CT scan. The degree of macroscopic resection was evaluated, comparing the pre/postoperative tumor volume and post-contrast enhancement characteristics. Tumor volume was calculated with the neuronavigation system software (Elements, Brainlab, Munich, Germany). The extent of resection (EOR) was classified as follows: total: 100%; near-total: >90%; and partial: <70%.

### 2.4. Intraoperative Technical Features

Intraoperative cortical and subcortical mapping was performed in all cases. A bipolar electrode (NIM—Eclipse NS—Medtronic 6743 Southpoint Drive N Jacksonville, USA) was used, with 1 mm electrodes and 0.5 mm distance between each one. The amplitude of the current was progressively increased stepwise by 1 mA until 2 mA was reached. We stimulated with biphasic square wave pulses of 1 ms at 60 Hz, with a maximum train duration of 4 s. Functional mapping was performed according to the preoperative planning related to the neuroimaging, using intraopeatively with the neuronavigation, and compared to the cortical stimulation responses. The marked areas were deemed inoperable. ViewSite-type MITRs (Brain Access System, Vycor Medical Inc. 951 Broken Sound Park Away, Suite 320, Boca Raton, USA) were used. In each case, the width, length and height of the MITRs used were recorded in the database. The chosen MITRs’ height depended on the distance between the outer table of the cranium and the superficial edge of the tumor.

### 2.5. Eight Step Strategy for PTA Design

**Figure 1:**
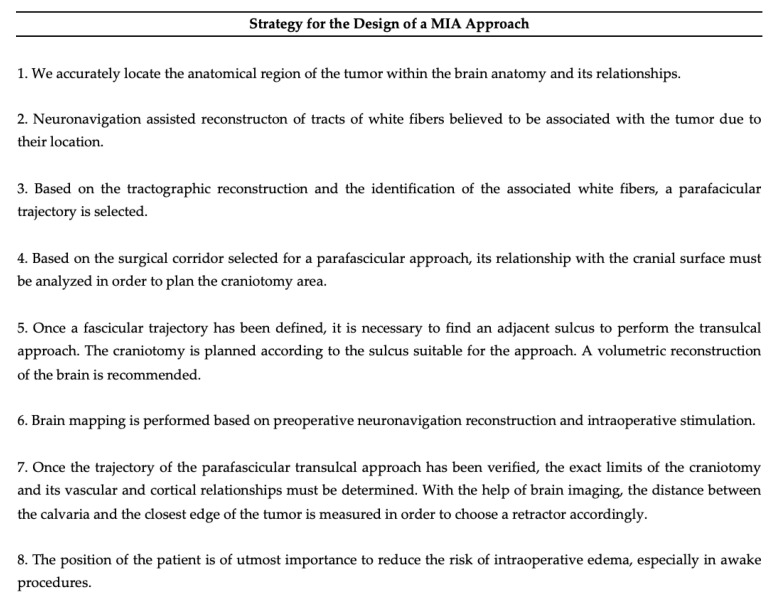
The Eight Step Strategy for PTA Design.

A strategy was standardized in eight steps for the approach using a minimally invasive technique: (1) determining the anatomical and functional location of the tumor, (2) tractography reconstruction, (3) determining the white fibers with potential risk of damage, (4) associating cortico-subcortical landmarks, (5) finding a suitable sulcus adjacent or within the surgical corridor, (6) designing the cortical and subcortical areas to be mapped, (7) designing the craniotomy and selecting the MITR and (8) checking the patient’s position (Figure 1).

## 3. Results

The results obtained in this study are presented in Table 1. A total of 72 operated-on patients were included in the study. The mean age of presentation of the disease in the group of patients was 49.6 years and the most affected gender was male (63.8%). At admission, neurological examination revealed paraphasia in 12.5% of the patients and only 11.1% presented some type of aphasia, described as follows: 2.7% presented anomic aphasia, 2.7% conduction aphasia, 1.3% global aphasia, 2.7% drill bit aphasia and 1.3% transcortical aphasia. Preoperatively, 41.6% had some motor deficit. Computerized campimetry was performed in 9.7% of the patients. Regarding the location of the tumor lesion, the anatomical region was mainly the frontal lobe (26.3%), followed by fronto-parietal (25%) and parietal (11.1%). The most frequent tumor type was HGG (34.72%), followed by LGG (17%), metastases (25%) and other lesions (11.1%). When evaluating the size of the incision, it was observed that the average of these was 5.58 cm, with 5 cm being the most frequent incision. In the craniectomies, it was estimated that the average diameter of these was 4.3 cm, with 4 cm being the most frequent diameter. Of the patients, 94.4% had a total gross resection. Postoperatively, 90.2% of the patients did not present new-onset deficits. Postoperative clinical outcome was evaluated in four aspects, postoperative KPS, long pathway damage, speech integrity and visual pathway integrity. Regarding postoperative KPS, 8.3% of patients had a KPS lower than admission postoperatively. Of the patients, 5.5% presented with impaired muscle strength in the limbs contralateral to the approach. Moreover, 4.1% presented some type of aphasia in the immediate postoperative period and none of the patients had visual impairment. Finally, we found that whenever the area of the tumor in an axial section was up to 5 times greater than the area of exposure of the MITRs, the tumor resection was greater than 90%. The most frequently used MITRs had the following dimensions: 17 mm wide, 11 mm high and 5 cm long.

## 4. Illustrative Cases

### 4.1. Case 1

A 29-year-old woman was referred to the emergency department with frequent headaches.

**Figure 2:**
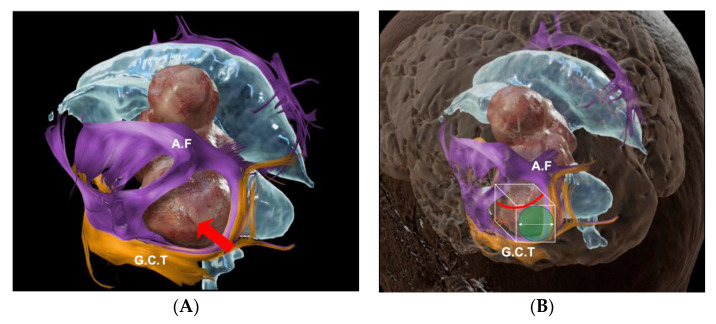
(**A**,**B**) show MRI rendering of a large intraventricular tumor (red arrow). Arcuate fasciculus (AF), geniculocalcarine tract (GTC) reconstruction helps in the planning stange for the resection of the tumor.

**Figure 3:**
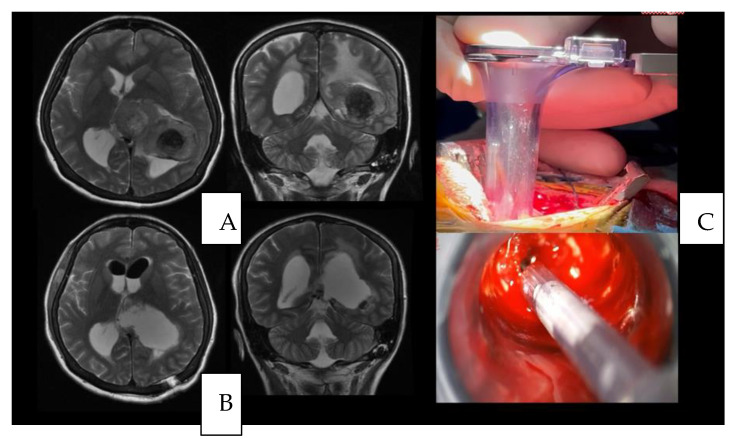
(**A**) Axial and coronal view of a preoperative T2 weighted MRI; (**B**) the postoperative axial and coronal view of a preoperative T2 weighted MRI that documents the GTR; (**C**) an intraoperative use of the tubular retractor.

The neurological examination upon admission to the hospital was unremarkable. She had a computerized campimetry without alterations. The head MRI with Gadolinium showed an interventricular tumor (Figure 2A,B). A safe GTR was performed (Figure 3). Histopathology was consistent with a WHO grade I meningioma.

### 4.2. Case 2

**Figure 4:**
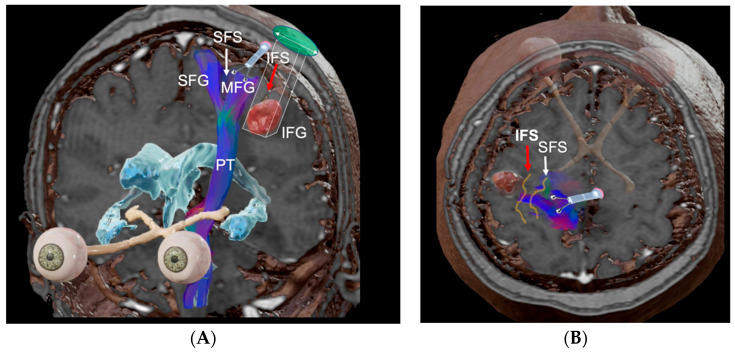
An intra-axial tumor at the junction of the inferior frontal gyrus and the pre-central sulcus. Fig (**A**) shows coronal view of merged T1 weighted MRI scan with corticospinal tract tractography. Fig (**B**) shows the axial view of the latter.

**Figure 5:**
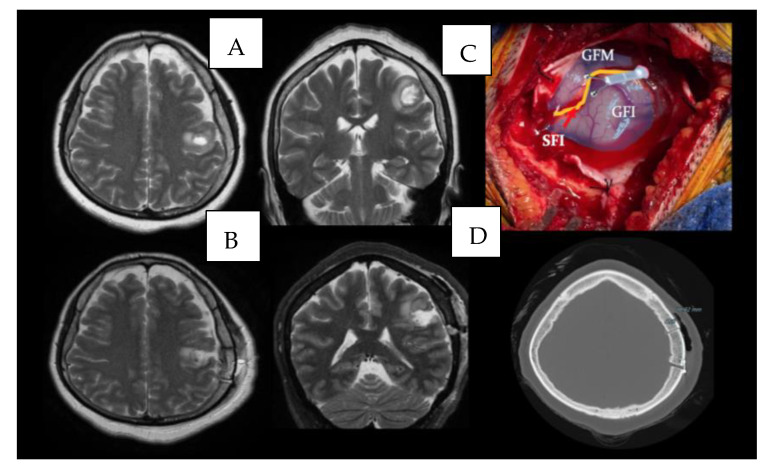
(**A**) Axial and coronal view of a preoperative T2 weighted MRI; (**B**) the postoperative axial and coronal view of a preoperative T2 weighted MRI that documents the GTR; (**C**) an intraoperative view of the cortical surface with the tracts highlighted; (**D**) the postoperative CT scan with bone algorithm that shows the small craniotomy.

A 46-year-old woman presented with seizures to the emergency department. The patient complained of dysarthria. An MRI was performed showing an intra-axial tumor at the junction of the inferior frontal gyrus and the pre-central sulcus (Figure 4A,B). The patient underwent resection and presented no postoperative neurological decline (Figure 5). Histopathological results were consistent with an HGG.

### 4.3. Surgical Nuances

Although there is no standardized technique for use of MITRs in brain surgery, the authors of this work believe that brain dissection can be performed with the help of the blunt tip of the MITRs. For an accurate and effective approach, MITRs must be guided with neuronavigation. Neurologists and electrophysiologists are essential for reliable intraoperative monitoring. After cortical stimulation, we recommend washing with cold solution to avoid triggering a seizure. Whenever possible, it is advisable to preserve the vascular, arterial and venous cortical and subcortical structures. In all cases, the MITRs should be fixed to a rigid support to avoid unintended movement that could lead to a change of the resection trajectory. Anesthetic monitoring should be carried out by anesthesiologists with extensive experience in neurological surgery and awake surgery.

## 5. Discussion

MIA in oncological neurosurgery remains controversial. Until two decades ago, a dogma of neuro-oncology was performing wide surgical approaches, which allowed the adequate management of intraoperative brain swelling, an effective control of bleeding, and a wide margin of maneuverability in case of complications, while achieving maximal EOR [2].

On the other hand, the PTA allows intracerebral access, while preserving the connectivity of the white matter tracts [11,12,13]. In this study, we described a surgical planning strategy for the design of a tubular PTA, guided by neuronavigation, for accurate cortical and subcortical mapping. The search for an ideal incision is mandatory, the traditional and best known techniques are based on the anatomy of the scalp, its vascularization. However, recently linear incisions have been adopted by some surgeons, considering their versatility, applicability and aesthetics [14]. In our work, in all cases, the incisions were linear, and even though no results were documented regarding the incidence of infections or aesthetic effects caused to the patients, we believe these allowed us to perform the craniotomy and the surgical resection of the lesion safely and widely, causing minor aesthetic damage to the skin.

The use of MIA in neurosurgery implies the planning of smaller craniotomies, permitting the brain to be approached in the same way as the prior existing ones [9]. Based on the multiple facts mentioned above, conservative neurosurgeons have given higher popularity to mini-craniotomies. However, even in other cranial pathologies, today it is becoming necessary to evaluate the benefits of brain approaches through these reduced cranial windows [15,16]. In our practice, the craniotomy was planned based on the relationship between the size of the tumor and the cranial area planned with neuronavigation [17]. This technique allowed us a safe approach and a complete resection or an NTR resection in most of the cases. Despite the fact that brain shift [18] and edema were not objectively evaluated, we presume that the small craniotomy openings allowed a lower dissociation curve with respect to the accuracy of navigation during the procedures and, in addition, can become a protective factor that prevents extra calvaria herniation.

**Figure 6:**
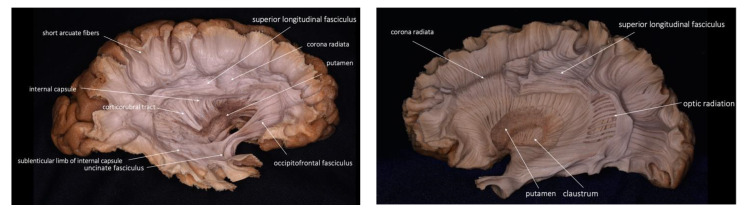
Cadaveric white matter dissection showing the fiber tracts commonly taken into account during the planning stage and intraoperatively for a safe tumor resection.

**Figure 7:**
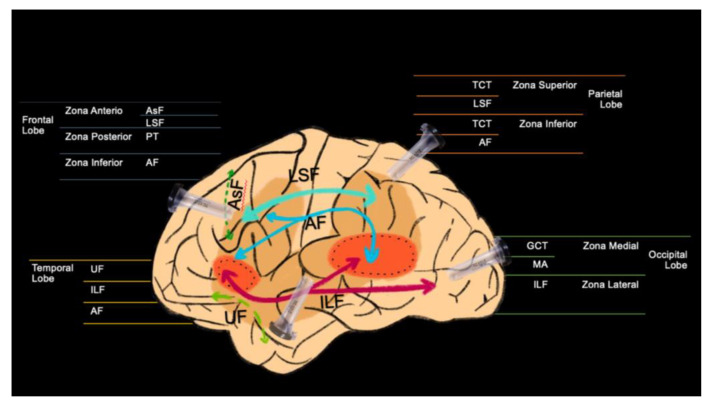
Fiber tracts usually reconstruct in our practice during preoperative planning. AsF: Aslant fasciculus; LSF: Superior Longitudinal fasciculus; PT: Pyramidal tract; AF: Arcuate fasciculus; UF: Uncinate fasciculus; ILF: Inferior longitudinal fasciculus; TCT: Thalamic cortical tract; GCT: Geniculo-calcarine tract; MA: Meyer’s loops.

PTA is widely described, even though its understanding requires a deep knowledge of anatomy and microsurgical skills, which allows the surgeon to travel through narrow corridors and deep within the brain tissue, causing limited damage to the fibers (Figure 6) [19]. Through the volumetric analysis of the cerebral cortex, its gyri and sulci, a cerebral sulcus was chosen in each patient far from the white fibers associated with the approach. We believe that depending on the location of the tumor, there are associated tracts that are mandatory to find in the reconstruction of the tractography in order to preserve their function (Figure 7).

The tubular MIA technique has been described in detail, as well as its benefits and utilities. This technique is associated with the use of neuronavigation, tractography and intraoperative monitoring, which makes it an ideal approach to prevent damage as much as possible, achieving an optimal resection rate versus neurological integrity [20,21,22,23]. A critical aspect that deserves attention is the preoperative assessment of the white matter tracts that will guide the intraoperative neuronavigation phase. In general, neuronavigation plays the role of allowing the surgeon to reach the lesion, but aiming to reduce the risk of neurological deficits by merging morphological data of the brain’s anatomy with the tractography will provide the safest trajectory to reach the tumor while sparing the fibers. In this context, the use of a tubular retractor perfectly suited the scope thanks to the shape of this tool. It follows that the tractography study is mandatory and paramount. In addition, the preoperative planning phase should be detailed and if possible much improved using the most modern technical tools including navigated trans-cranial magnetic stimulation (nTMS) [24,25,26,27]. nTMS have been proved to improve the reliability of the brain mapping and offers the opportunity to simulate the resection and related risk of deficit by causing a transitory lesion that can be localized, archived and merged with the dataset of the patient to be used during surgery. In this setting, nTMS adds awake information to sleeping surgical procedures.

Despite that the results presented in our study support the use of MIA, it is necessary to continue developing strategies to evaluate other aspects, allowing one to solve the questions of the critics of this tool. We are convinced that MIAs offer to modern neurosurgery the possibility of mutating some practices, in specific cases, which ends up influencing the clinical and surgical results of patients, as has already happened in other surgical specialties.

### Limitations

Our study deals with an emerging trend in minimally invasive surgery for brain tumors. However, it does show several weaknesses. First, it is not a comparative study but rather a prospective observational study with its inherent biases. However, we report 9.8% of new-onset deficits that we consider acceptable based on the eloquence of the localization of the tumor. Secondly, the heterogeneity of the population. This may play a role in the clinical outcome of the procedure, but the goal of our study is to describe the steps we found helpful in the planning phase for MITRs. Third, we did not use nTMS in the planning stage. nTMS could provide useful information that could further reduce the risk of neuro-deficit and its use should be investigated as a potential new point of the strategy presented here in future studies.

## 6. Conclusions

We suggest considering adding MIA in surgical routine planning to perform safe tubular PTA for eloquent area tumors, which, added to the implementation of technologies such as intraoperative cortical and subcortical mapping, as well as tractography reconstruction, may improve maximal safe resection, preserving neurological function. Despite our results, there are currently no studies evaluating the benefits of this procedure compared to conventional techniques, and further studies are needed to evaluate the best indications for each one.

## Figures and Tables

**Table 1:** Tumor characteristics and related clinical outcome.

Neurological Examination		%
Altered Force		41.6
Language Disturbances		
Paraphasia		12.5
Aphasia		11.1
	Anomic	2.7
	Conduction	2.7
	Global	1.3
	Broca’s	2.7
	Transcortical	1.3
Tumor Localization		
Frontal		26.3
Parietal		11.1
Frontoparietal		25
Other		37.6
Tumor Type		
High Grade Gliomas		34.7
Low Grade Gliomas		29.2
Metastasis		25
Other		11.1
Resection		
Total		94.4
Sub-Total		5.5
Partial		0

## Data Availability

Not applicable.

## Abbreviations

MIA	minimally invasive approach (MIA)
MITRs	minimally invasive tubular retractors (MITRs)
PTA	parafascicular transulcal approach (PTA)
KPS	Karnofsky Performance Scale (KPS)
GCS	Glasgow Coma Scale (GSC)
MRC	Medical Research Council (MRC)
LGG	low-grade glioma (LGG)
HGG	high-grade glioma (HGG)
MRI	magnetic resonance imaging (MRI)
DTI	diffusion tensor imaging (DTI)
SLF	superior longitudinal fasciculus (SLF)
ILF	inferior longitudinal fasciculus (ILF)
GCT	geniculo-calcarine fasciculus (GCT)
UF	uncinate fasciculus (UF)
CST	corticospinal tract (CST)
AF	arcuate fasciculus (AF)
AsF	anterior superior frontal (AsF)
nTMS	navigated trans-cranial magnetic stimulation (nTMS)
